# Activation of the Nrf2/HO-1 Pathway by *Amomum villosum* Extract Suppresses LPS-Induced Oxidative Stress *In Vitro* and *Ex Vivo*

**DOI:** 10.1155/2020/2837853

**Published:** 2020-05-03

**Authors:** Dong-Woo Lim, Hee-Jin Choi, Sun-Dong Park, Hyuck Kim, Ga-Ram Yu, Jai-Eun Kim, Won-Hwan Park

**Affiliations:** ^1^Department of Pathology, College of Korean Medicine, Dongguk University, Goyang-si 10326, Republic of Korea; ^2^Department of Korean Medical Science, College of Korean Medicine, Pusan National University, Yangsan-si 50612, Republic of Korea; ^3^Department of Prescription, College of Korean Medicine, Dongguk University, Goyang-si 10326, Republic of Korea; ^4^Institute of Korean Medicine, Dongguk University, 32 Dongguk-ro, Goyang-si 10326, Republic of Korea; ^5^Department of Diagnostics, College of Korean Medicine, Dongguk University, Dongguk-ro 32, Goyang-si 10326, Republic of Korea

## Abstract

Despite its deleterious effects on living cells, oxidative stress plays essential roles in normal physiological processes and provides signaling molecules for cell growth, differentiation, and inflammation. Macrophages are equipped with antioxidant mechanisms to cope with intracellular ROS produced during immune response, and Nrf2 (NF-E2-related factor 2)/HO-1 (heme oxygenase-1) pathway is an attractive target due to its protective effect against ROS-induced cell damage in inflamed macrophages. We investigated the effects of ethanol extract of *A. villosum* (AVEE) on lipopolysaccharide- (LPS-) stimulated inflammatory responses generated via the Nrf2/HO-1 signaling pathway in murine peritoneal macrophages and RAW 264.7 cells. AVEE was found to suppress the NF-*κ*B signaling pathway, thus, to reduce proinflammatory cytokine, nitric oxide, and prostaglandin levels in peritoneal macrophages and Raw 264.7 cells treated with LPS, and to enhance HO-1 expression by activating Nrf2 signaling. Furthermore, these anti-inflammatory effects of AVEE were diminished when cells were pretreated with SnPP (a HO-1 inhibitor). HPLC analysis revealed AVEE contained quercetin, a possible activator of the Nrf2/HO-1 pathway. These results show *A. villosum* ethanol extract exerts anti-inflammatory effects by activating the Nrf2/HO-1 pathway in LPS-stimulated macrophages.

## 1. Introduction

Oxidative stress can be defined as an imbalance surfeit of ROS (reactive oxygen species) production as compared with ROS depletion by antioxidant defense systems [1]. Despite their deleterious effects, ROS play crucial roles in the physiological processes and metabolisms of organisms that utilize oxygen [2, 3]. At the physiological level, ROS act as key signaling molecules for cell growth, differentiation, and inflammation [4]. However, when ROS levels are excessive, they cause oxidative damage to biomolecules such as lipids, proteins, and DNA [5] and can induce metabolic dysfunctions and apoptosis [6].

Inflammation is complex physiological response to noxious stimuli, such as physical or chemical injuries, or infections [7]. Excessive ROS induced by, for example, lead, carbon tetrachloride (CCl_4_), or LPS can activate MAPK and NF-*κ*B pathways and initiate inflammatory responses [8–11]. Of these ROS inducing agents, LPS has been intensively studied in the context of inflammatory response progression in macrophages [12].

Resident macrophages are activated after stimulation of toll-like receptors (TLRs), which act as primary LPS receptors, and, when activated, TLRs signal proinflammatory cytokine production, and hence, initiating inflammation [13]. ROS production also occurs during phagocytosis [14] and macrophage differentiation [15] and increases in intracellular ROS caused by respiratory burst during phagocytosis can be lethal to macrophages and cause apoptosis [16]. However, macrophages are equipped with antioxidant enzymes to counter endogenous ROS production. Accordingly, inflammation, oxidative stress, and the expressional statuses of antioxidant enzymes are closely interrelated [17].

Nrf2 (NF-E2-related-factor-2) is a representative, redox-sensitive transcription factor that controls the expressions of antioxidant enzymes such as NADPH, quinone oxidoreductase (NQO1), glutamate cysteine ligase (GCL), glutathione S-transferase (GST), catalase (CAT), and heme oxygenase-1 (HO-1) [18–20] and its functions and those of its downstream genes have been intensively studied in the context of oxidative stress and chemically induced cellular damage [21].

HO-1 is induced by oxidative and inflammatory signals in most cells [22]. HO-1 catalyzes the degradation of heme to produce carbon monoxide, free iron, and biliverdin [23], the latter of which is converted into bilirubin, which is largely responsible for the protective effects of HO-1 [24]. The regulation of HO-1 expression is now regarded a therapeutic target for several diseases [25], and in one study conducted using HO-1 deficient mouse models, it was found that HO-1 fundamentally modulates early inflammatory response [26]. Therefore, concerted efforts are being made to discover natural product-derived modulators of the Nrf2/HO-1 pathway to conquer chronic inflammatory diseases [27].

*Amomum villosum* Lour. (*A. villosum*) belongs to the Zingiberaceae family and is used to treat gastrointestinal disorders and nausea in traditional Chinese medicine [28, 29]. Previous studies have shown *A. villosum* has analgesic, anti-inflammatory [30], and antimicrobial [31] properties and ameliorates anti-nonalcoholic fatty liver disease (NAFLD) [28]. Furthermore, the high volatile oil and polysaccharide contents of *A. villosum* suggest it probably has other undiscovered pharmacologic effects. However, its effects on murine macrophages have not been investigated. In the present study, we investigated the effects of an ethanol extract of *A. villosum* (AVEE) on LPS-stimulated murine peritoneal macrophages and Raw 264.7 cells and mechanisms responsible for these effects.

## 2. Materials and Methods

### 2.1. Reagents and Animals

Dulbecco's Modified Eagle Medium (DMEM), fetal bovine serum (FBS), and penicillin/streptomycin solution were purchased from Invitrogen (Carlsbad, CA, USA). Primary antibodies for iNOS, COX-2, HO-1, Nrf2, p65, I*κ*B-*α*, p-I*κ*B-*α*, *β*-actin, and lamin B were purchased from Santa Cruz (Santa Cruz, CA, USA). ELISA kits for IL1*β*, TNF-*α*, and PGE_2_ were purchased from R&D Systems (Minneapolis, MN, USA). *Escherichia coli* lipopolysaccharide (LPS), dimethyl sulfoxide (DMSO), and Griess reagents were purchased from Sigma Aldrich Corp. (St Louis, MO, USA). Tin protoporphyrin IX dichloride (SnPP IX, a HO-1 inhibitor) and cobalt protoporphyrin (CoPP, a HO-1 inducer) were obtained from Porphyrin Products (Logan, UT, USA). Radioimmunoprecipitation assay (RIPA) buffer and a NE-PER nuclear extraction kit were purchased from Thermo Scientifics (Waltham, MA, USA). A TransAM kit, which was used to measure the DNA-binding activity of NF-*κ*B, was obtained from Active Motif (Carlsbad, CA, USA). A Bio-Rad Protein Assay kit (based on the Bradford method) used for determining protein concentrations was purchased from Bio-Rad Laboratories (Hercules, CA, USA).

Peritoneal macrophages were isolated from 10-week old C57/BL6J mice using 4% thioglycollate medium. Briefly, thioglycollate (1 mL) was injected intraperitoneally 96 h before sacrifice. Mice were anesthetized with mixture of Zoletil and Rompun, injected intraperitoneally with 10 mL of RPMI, and later, 6–8 mL of peritoneal fluid was withdrawn using a syringe. The fluid was then centrifuged to separate cells from debris. The cells obtained were incubated for 3 h at 37°C and washed with Dulbecco's Phosphate-Buffered Saline (DPBS), and adherent macrophages were collected for further study. All protocols for animal experiments were approved beforehand by the ethics committee of Dongguk University (IACUC-2016-055).

### 2.2. Preparation of AVEE

The fruits of *Amomum villosum* (Zingiberaceae) were purchased from the Dongguk University Hospital Herbal Drugstore (Ilsan, Republic of Korea); a voucher specimen was deposited at the College of Korean Medicine at Dongguk University. Fruits (50 g) were extracted two times with 70% ethanol (1 L) for 2 h at 95°C, and the extract obtained was passed through Whatman #2 filter paper. Evaporation of the filtrate *in vacuo* resulted in a 70% ethanol extract (6.8 g, 13.6 w/w %), which was then suspended in distilled water (100 mL), filtered, and dried *in vacuo*. The residue obtained was dissolved in hot ethanol, filtered, and reevaporated to obtain AVEE (660 mg, 1.32 w/w %). For experiments, AVEE was dissolved in DMSO (dimethyl sulfoxide) at a concentration of 0.05%. Supportive studies showed that, at the concentrations used, DMSO had no effect on cell viabilities.

### 2.3. Cell Viability Assay

Mouse peritoneal macrophages and RAW 264.7 cells (a macrophage cell-line; American Type Culture Collection) were maintained in FBS-supplemented (10%, v/v) DMEM, 100 U/mL penicillin, and 100 *μ*g/mL streptomycin. Cells were incubated in a 5% CO_2_ humidified atmosphere at 37°C. Cell viabilities were measured using an MTT assay. Briefly, mouse peritoneal macrophages and RAW 264.7 cells were plated at a concentration of 2 × 10^5^ cells per well in 24-well plates and treated with different concentrations of AVEE for 24 h. Supernatants were discarded and 0.5 mg/mL of MTT reagent was added. After 4 h of incubation at 37°C under 5% CO_2_, supernatants were removed and DMSO was added to dissolve the formazan crystals formed in live cells. Optical densities were measured at 540 nm using an ELISA plate reader.

### 2.4. Determination of the Expression of Inflammatory Cytokine Genes in Peritoneal Macrophages

Isolated murine macrophages were seeded in a 6-well plate, at 5 × 10^5^ cells per well, preincubated with AVEE for 30 min, and treated with LPS (1 *μ*g/mL) for 24 h. Intact mRNA was manually isolated using Trizol reagent (Thermo Fisher, Waltham, USA) according to the manufacturer's instructions and used to synthesize cDNA. Quantitative real-time PCR was conducted using SYBR green fluorescence dye over 45 amplification cycles consisting of denaturation at 95°C for 10 s, annealing at 55°C-58°C for 15 s, and extension at 72°C for 15 s. The following primers were used: IL-1*β*—forward, 5′-GCCCATCCTCTGTGACTCAT-3′, reverse, 5′-AGGCCACAGCTATTTTGTCG-3′; IL-6—forward, 5′-AGTTGCCTTCTTGGGACTGA-3′, reverse, 5′-CAGAATTGCCATTGCACAAC-3′; TNF-*α*—forward, 5′-GAACTGGCAGAAGAGGCAG-3′, reverse, 5′-AGGGTCTGGGCCATAGAACT-3′; COX-2—forward, 5′-AGAAGGAAATGGCTGCAGAA-3′, reverse, 5′-GCTCGGCTTCCAGTATTGAG-3′; and beta-actin (*β*-actin)—forward, 5′-GCAAGTGCTTCTAGGCGGAC-3′, reverse; 5′-AAGAAAGGGTGTAAAACGCAGC-3′ used as the internal control. Ct results with a melting curve were checked using Roche LightCycler 480 software (Roche Applied Science, USA). Ct values for the expression of each cytokine gene were normalized using Ct values of *β*-actin gene expression.

### 2.5. Measurement of Nitric Oxide Production

NO production was measured using Griess reagent. Briefly, RAW 264.7 cells were seeded at 2 × 10^5^ cells per well in 24-well plates and treated with AVEE with or without SnPP IX plus LPS for 18 h. Next, 100 *μ*L aliquots of media were mixed with 100 *μ*L Griess reagent (0.1% *N*-(1-naphthyl)ethylenediamine and 1% sulfanilamide in 5% phosphoric acid), and 15 min later absorbances were measured at 540 nm using a microplate reader (VersaMax, Molecular Devices, CA, USA).

### 2.6. Measurement of PGE2, TNF-*α*, and IL-1*β* Levels in Culture Media

Levels of PGE_2_, TNF-*α*, and IL-1*β* in culture media were measured using an ELISA kit. Briefly, RAW 264.7 cells were seeded at 2 × 10^5^ per well in 24-well plates and treated with AVEE with or without SnPP IX plus LPS for 18 h. Culture media were then collected and the concentrations of PGE2, TNF-*α*, and IL-1*β* were measured using an ELISA kit.

### 2.7. Preparation of Nuclear and Cytosolic Fractions

RAW 264.7 cells were seeded at 2 × 10^6^ cells per dish in 60 mm culture dishes and treated with various concentrations of AVEE and LPS for 1 h. Nuclear and cytosolic proteins were isolated using an NE-PER nuclear extraction kit.

### 2.8. Western Blot Analysis

Whole proteins were isolated using RIPA buffer and quantified using the Bradford method. Equal amounts of proteins were separated by SDS-PAGE (10%) and transferred to nitrocellulose membrane, which were then blocked with 5% skim milk, incubated with primary antibodies at 4°C overnight, washed three times, and incubated with secondary antibodies at 4°C for 1 h. Protein was detected using a Fusion Solo chemiluminescence system (Vilber Lourmat, Marne-la-Vallée, France) and analyzed using Bio-1D advanced software.

### 2.9. DNA-Binding Activity of NF-*κ*B

RAW 264.7 cells were seeded at 2 × 10^6^ cells per dish in 60 mm culture dishes and treated with various concentrations of AVEE and LPS for 1 h. DNA-binding activity of NF-*κ*B was measured using a TransAM kit according to manufacturer's instructions. Briefly, 20 *μ*g of nuclear proteins was diluted in complete lysis buffer (1 M DTT, protease inhibitor cocktail, lysis buffer AM2) and added to wells with 30 *μ*L of complete binding buffer (1 M DTT, herring sperm DNA, binding buffer AM3). Plates were incubated for 1 h with agitation (100 rpm) and washed three times, and then 100 *μ*L of NF-*κ*B antibody (1 : 1000 dilution) was added and samples were incubated for an additional 1 h with mild agitation. After washing each well three times, 100 *μ*L of HRP-conjugated antibody (1 : 1000 dilution) was added, and plates were incubated for 1 h with agitation. Wells were then washed four times, 100 *μ*L of developing solution was added to each well, kept for 5 min in the dark. Finally, stop solution was added (100 *μ*L/well) and absorbance was read on a spectrophotometer at 450 nm.

### 2.10. Immunofluorescence Assay

RAW 264.7 cells were seeded onto Lab-Tek II chamber slides and incubated for 24 h. Various concentrations of AVEE were then added with LPS and incubated for 1 h. Cells were then fixed in formalin, blocked with 1% bovine serum albumin in PBS for 30 min, treated with primary antibody diluted 1 : 200 in PBS overnight at 4°C, and incubated with fluorescein isothiocyanate- (FITC-) labeled secondary antibody diluted 1 : 2000 in PBS for 1 h. To detect nuclei, cells were treated with 1 *μ*g/mL of DAPI for 5 min, washed three times with PBS, and mounted on chamber slides using Dako Fluorescent mounting medium. Stained cells were observed and photographed using a fluorescence microscope (BX50, Olympus, Tokyo, Japan).

### 2.11. High Performance Liquid Chromatography (HPLC) of AVEE

The HPLC unit used was SNKNM series instrument equipped with a sample injector and a diode-array UV/VIS detector (DAD) (SYKAM, Germany). An Inertsil ODS analytical C-18 column (4.6 × 150 nm; 5 µm, GL Science Inc., Tokyo, Japan) was used, with an injection volume of 20 *µ*L. The mobile phase comprised water (A) and methanol (B), with an applied linear gradient of 10% increasing to 100% B within 40 min. The column was prewashed with 10% B for 20 min, after which the condition maintained 20 min for equilibration. The flow rate was adjusted to 0.7 mL/min, and the detection was adjusted to 254 nm wavelength.

### 2.12. Data Analysis

Results are presented as the means ± standard deviation of at least three independent experiments performed in triplicate. Data was analyzed using One-Way ANOVA with Dunnett's multiple comparison tests. *P* values of <0.05, <0.01, or <0.005 were considered significant, as indicated.

## 3. Results

### 3.1. Effect of AVEE on RAW 264.7 Cell Viability

**Figure 1:**
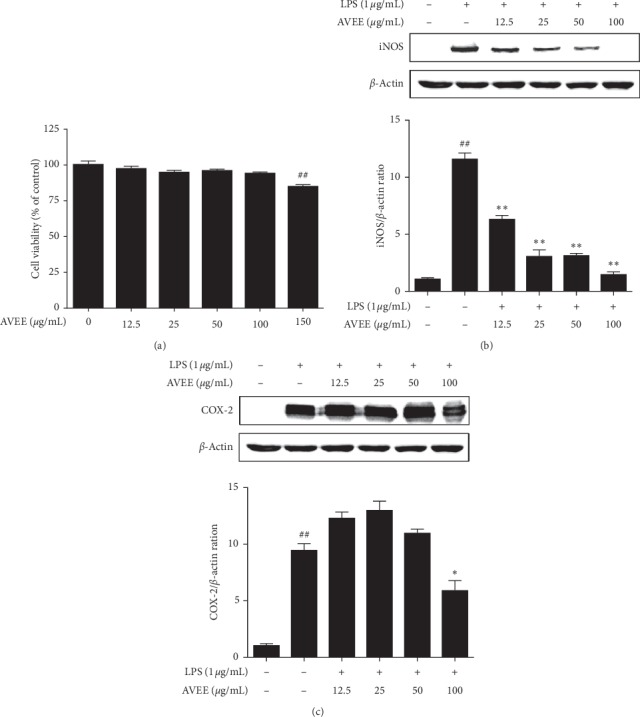
Effects of AVEE on RAW 264.7 cell viability and on the LPS-induced expressions of proinflammatory enzymes. RAW 264.7 cells were incubated for 24 h in the presence of various concentrations of AVEE (12.5, 25, 50, 100, or 150 *μ*g/mL). Cell viabilities were measured using an MTT assay (a). Cells were pretreated with various concentrations of AVEE (12.5, 25, 50, or 100 *μ*g/mL) 3 h before LPS stimulation and then incubated for a further 18 h in the presence of LPS (1 *μ*g/mL). Protein levels of iNOS (b) and COX-2 (c) were detected by western blotting. Results are presented as the means ± SDs of three independent experiments. ^##^*P* < 0.01 versus treatment naïve controls. ^*∗*^*P* < 0.05 and ^*∗∗*^*P* < 0.01 versus LPS treated controls.

To determine the optimal AVEE concentration, its effects on RAW 264.7 cell viability were assessed using the MTT assay (Figure 1(a)). Cells were incubated with various concentrations of AVEE (12.5, 25, 50, 100, and 150 *μ*g/mL) for 24 h. No significant difference in cell viability was observed up to a concentration of 100 *μ*g/mL (94.0% of the control), and thus AVEE concentrations of 12.5, 25, 50, or 100 *μ*g/mL were used in subsequent experiments.

### 3.2. AVEE Inhibited iNOS and COX-2 Expressions in Inflamed RAW 264.7 Cells

The influence of AVEE on the protein expressions of iNOS and COX-2 was investigated in LPS-stimulated macrophages. RAW 264.7 cells were preincubated with AVEE before being treated with LPS. As was expected, immunoblotting showed LPS markedly increased iNOS and COX-2 protein levels. AVEE significantly and dose-dependently inhibited the expression of iNOS (Figure 1(b)) and obviously suppressed COX-2 levels at 100 *μ*g/mL (Figure 1(c)).

### 3.3. AVEE Reduced the LPS-Induced Productions of NO, PGE_2_, and Inflammatory Cytokines in RAW 264.7 Cells

**Figure 2:**
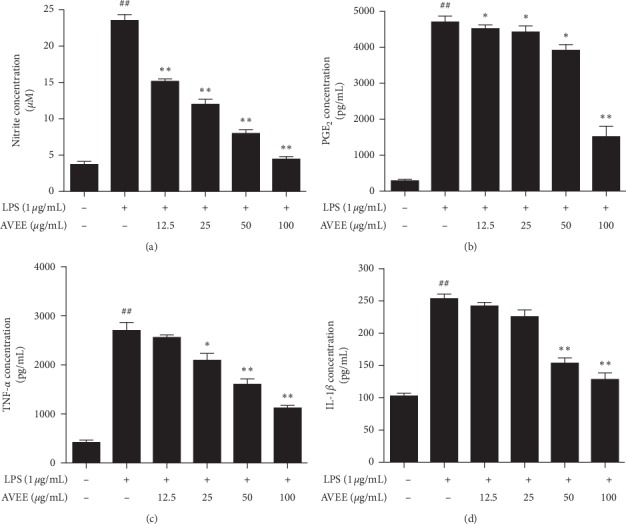
Effects of AVEE on the inductions of NO and inflammatory cytokines by LPS. RAW 264.7 cells were pretreated with the various concentrations of AVEE (12.5, 25, 50, or 100 *μ*g/mL) 3 h before LPS stimulation, and then for a further 18 h in the presence of LPS (1 *μ*g/mL). No concentrations were measured using a Griess reagent system (a). PGE_2_ (b), TNF-*α* (c), and IL-1*β* (d) expressions were quantified by ELISA as described in Materials and Methods. Results are presented as the means ± SDs of three independent experiments. ^##^*P* < 0.01 versus treatment naïve controls. ^*∗*^*P* < 0.05 and ^*∗∗*^*P* < 0.01 versus LPS controls.

LPS-induced increases in NO and PGE_2_ (Figures 2(a) and 2(b)) levels were reduced by AVEE pretreatment. Furthermore, ELISA showed AVEE dose-dependently suppressed LPS induced increases in the levels of TNF-*α* and IL-1 *β* (Figures 2(c) and 2(d)).

### 3.4. AVEE Inhibited NF-*κ*B Signal Activation in RAW 264.7 Cells

**Figure 3:**
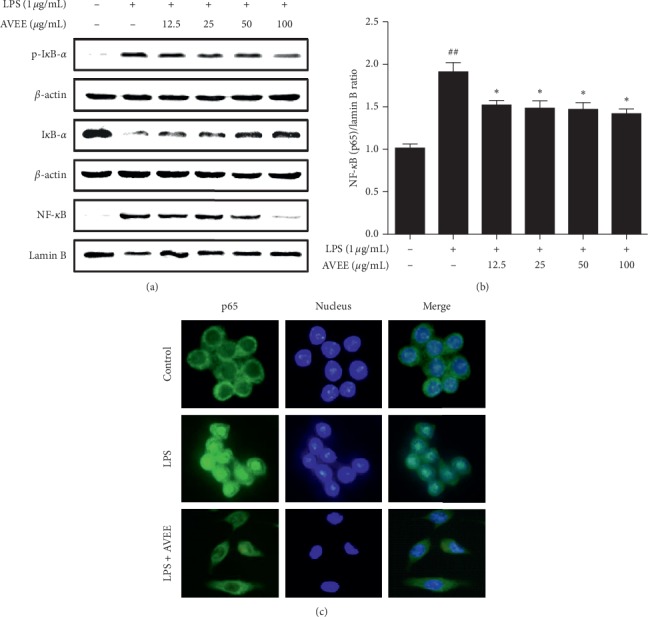
Effects of AVEE on LPS-induced NF-*κ*B pathway activation. RAW 264.7 cells were treated with various concentrations of AVEE (12.5, 25, 50, or 100 *μ*g/mL) for 3 h and stimulated with LPS (1 *μ*g/mL) for another 1 h. Cytosolic I*κ*B phosphorylation and NF-*κ*B translocation to nucleosomes were detected by Western blotting (a). NF-*κ*B DNA-binding activity was assessed using a TransAM kit (b). Nuclear NF-*κ*B translocation was visualized under an immunofluorescence microscope (c). Results are presented as the means ± SDs of three independent experiments. ^##^*P* < 0.01 versus treatment naïve controls. ^*∗*^*P* < 0.05 versus LPS controls.

We also examined the effects of AVEE on NF-*κ*B translocation and I*κ*B-*α* phosphorylation in RAW 264.7 cells using immunoblot, TransAM kit, and immunofluorescence images. AVEE dose-dependently inhibited the nuclear translocation of NF-*κ*B (Figure 3(a)) and suppressed LPS-induced cytoplasmic I*κ*B-*α* degradation and phosphorylation. Moreover, AVEE also suppressed LPS-induced NF-*κ*B DNA-binding activity (Figure 3(b)). These results were confirmed by immunofluorescence microscopic images (Figure 3(c)).

### 3.5. AVEE Induced HO-1 Expression and the Nuclear Translocation of Nrf2 in RAW 264.7 Cells

**Figure 4:**
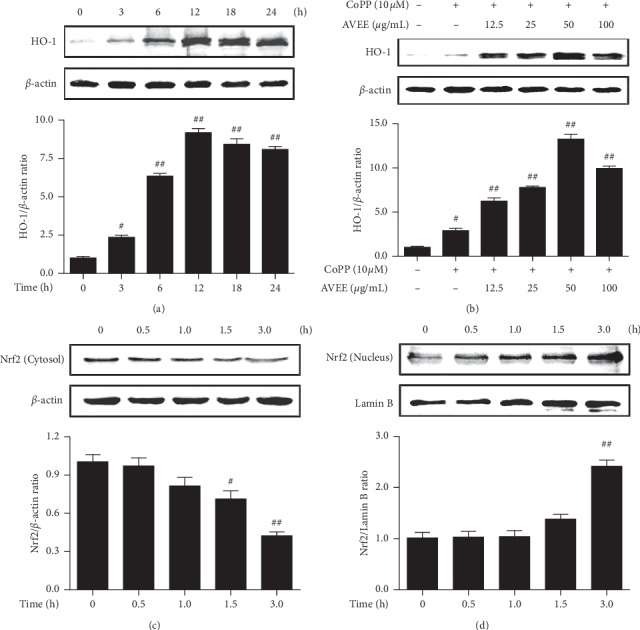
Effects of AVEE on HO-1 expression and the nuclear translocation of Nrf2. RAW264.7 cells were incubated with 100 *μ*g/mL of AVEE for indicated times and HO-1 expression was examined by western blot analysis (a). RAW 264.7 cells were treated with various concentrations of AVEE (12.5, 25, 50, and 100 *μ*g/mL) for 18 h CoPP (10 *µ*M; a HO-1 inducer) was used as the positive control (b). Nrf2 expression levels of in cytoplasm (c) and nucleus (d) were assessed by Western blotting. Columns are the means ± SDs of independent experiments. ^#^*P* < 0.05 and ^##^*P* < 0.01 versus treatment naïve controls.

The induction of HO-1 expression was monitored after treating RAW 264.7 cells with AVEE. The expression of HO-1 was apparent after 3 h of treatment and peaked at 12 h in the presence of 100 *μ*g/mL AVEE (Figure 4(a)). And we confirmed that AVEE increased HO-1 expression in combination with 10 *μ*M CoPP (a HO-1 inducer) (Figure 4(b)). Furthermore, immunoblotting showed obvious changes in the localization of Nrf2 from cytoplasm to nuclei after 3 h of AVEE treatment (100 *μ*g/mL) (Figure 4(c)). The results indicate AVEE treatment promoted the nuclear translocation of Nrf2 and activated the Nrf2/HO-1 pathway to increase HO-1 level (Figure 4(d)).

### 3.6. AVEE Suppressed NO, PGE_2_, and Proinflammatory Cytokine Levels by Inducing HO-1 Expression

**Figure 5:**
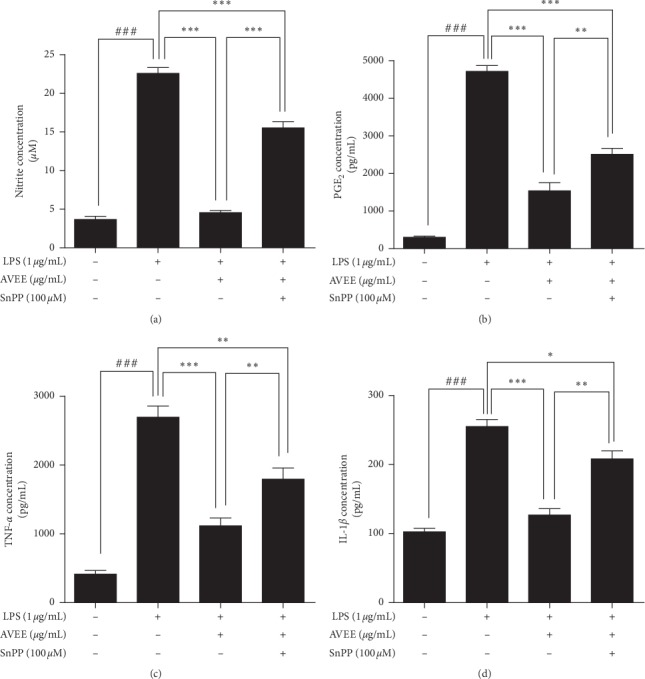
Effects of AVEE on SnPP-induced proinflammatory enzyme and cytokine levels. RAW 264.7 cells were incubated with SnPP (an HO-1 inhibitor) for 1 h treated with AVEE at 100 *μ*g/mL for 3 h and stimulated with LPS (1 *μ*g/mL). NO (a), PGE_2_ (b), TNF-*α* (c), and IL-1*β* (d) levels were assessed by ELISA as described in Materials and Methods. Results are presented as the means ± SDs of three independent experiments. ^###^*P* < 0.005 versus treatment naïve controls. ^*∗∗*^*P* < 0.01 and ^*∗∗∗*^*P* < 0.005 versus the indicated group.

We investigated whether HO-1 inhibition influences the inhibitory effects of AVEE on LPS-stimulation, which was observed in result above. The inhibitory effects of AVEE pretreatment on the LPS-induced upregulations of NO, PGE2, TNF-*α*, and IL-1*β* levels in RAW 264.7 were found to be significantly decreased by treating cells with 100 *μ*M of SnPP for 1 h before AVEE pretreatment (Figures 5(a)–5(d)).

### 3.7. AVEE Inhibited Proinflammatory Gene Expression in Mouse Peritoneal Macrophages

**Figure 6:**
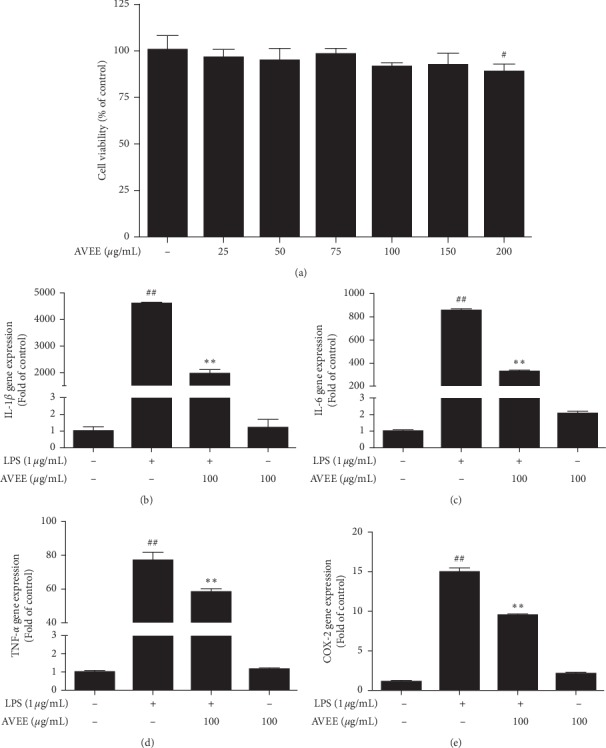
Effects of AVEE on proinflammatory gene expression in murine peritoneal macrophages. Murine macrophages were incubated with various concentrations of AVEE (25, 50, 75, 100, 150, and 200 *μ*g/mL) for 24 h. Cell viabilities were measured using an MTT assay (a). Isolated peritoneal murine macrophages were incubated with AVEE (100 *μ*g/mL) for 3 h and stimulated with LPS (1 *μ*g/mL). IL-1*β* (b), IL-6 (c), COX-2 (d), and TNF-*α* (e) mRNA expressions are presented as fold increases versus treatment naïve controls. Results are presented as the means ± SDs of three independent experiments. ^##^*P* < 0.01 versus treatment naïve controls. ^*∗∗*^*P* < 0.01 versus LPS treated controls.

Isolated murine macrophages were incubated with or without AVEE for 24 h to determine the effect of AVEE treatment on cell viability. As was observed for RAW 264.7 cells, AVEE did not significantly affect viability at concentrations of <100 *μ*g/mL, but at 200 *μ*g/mL, AVEE did significantly reduce cell viability (Figure 6(a)). Murine macrophages stimulated with 1 *μ*g/mL LPS showed marked increases in the mRNA expressions of IL-6, IL-1*β*, COX-2, and TNF-*α*, but AVEE pretreatment (100 *μ*g/mL) prevented these upregulations. When treatment naïve macrophages were treated with AVEE, the expressions of these inflammatory genes increased slightly (Figures 6(b)–6(e)).

### 3.8. HPLC Analysis of AVEE

**Figure 7:**
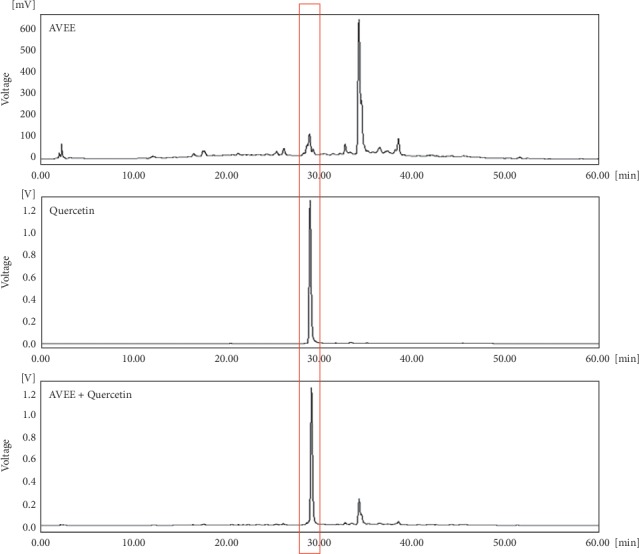
HPLC chromatogram of AVEE.

AVEE was analyzed by HPLC using a detection wavelength of 254 nm. Three samples (AVEE, quercetin, and AVEE + quercetin) were run to enable accurate retention time comparisons. The results obtained showed AVEE contained quercetin, which is a known bioactive component of *A. villosum* (Figure 7) [32].

## 4. Discussion

The present study shows AVEE suppresses LPS-induced inflammatory response by suppressing oxidative stress via the Nrf2/HO-1 pathway. Macrophages of the murine peritoneal cavity have been widely used to investigate anti-inflammatory effects and mechanisms of natural products [33, 34]. LPS triggers macrophage activation through TLR4, and this activation results in the explosive production of proinflammatory cytokines [35]. In particular, excessive nitric oxide (NO) functions as proinflammatory mediator [36] and confers on macrophages the ability to kill invading pathogens [37]. In addition, the upregulation of inducible cyclooxygenase (COX-2) results in the inductions of various tissue-specific isomeric prostaglandins [38]. Furthermore, these proinflammatory markers are generally elevated in classically activated (M1) macrophages [39].

As was expected, LPS elicited inflammatory responses in macrophages and markedly increased NO and PGE_2_, TNF*α*, IL-1*β*, iNOS, and COX-2 levels, and all were significantly and dose-dependently reduced by pretreating cells with AVEE. NF-*κ*B is a well-known p50/RelA (p65) heterodimeric transcription factor that is responsible for the expressions of various proinflammatory genes encoding cytokines and chemokines [40]. The DNA-binding activity of NF-*κ*B is modulated by I*κ*B phosphorylation and degradation, which follow the activation of I*κ*B kinase (IKK) complex [41]. In the present study, AVEE pretreatment blocked LPS-induced I*κ*B phosphorylation and inhibited the translocation of NF-*κ*B in murine macrophages, as evidenced by immunoblot and immunofluorescence images, which indicated AVEE suppressed LPS-induced inflammatory responses by blocking the NF-*κ*B pathway.

LPS-stimulated macrophages create excessive oxidative stress and produce nitric oxide, which can activate upstream kinases of NF-*κ*B such as IKK and Akt [42, 43]. The importance of HO-1 expression in the maintenance of redox balance was demonstrated in a study on HO-1 knockout mice, which exhibited high levels of ROS, proinflammatory cytokines, and oxidized LDL in macrophages [44] and, thus, demonstrated that HO-1 importantly protects macrophages from ROS [45] by inhibiting NF-*κ*B signaling and inflammatory responses. Furthermore, HO-1 expression is preferentially expressed in M2 macrophages and causes a shift from an inflammatory to an anti-inflammatory state and, thus, facilitates the resolution of inflammatory processes [46].

In the following experiment, AVEE notably induced HO-1 expression in presence of 10 *μ*M CoPP and induced the nuclear localization of Nrf2, which is responsible for upregulation of cellular antioxidant defense mechanisms. Clearer evidences came from the assay with SnPP, which is inhibitor of HO-1, when SnPP treatment markedly depressed effect of AVEE against LPS. For example, suppressions of LPS-induced increases in inflammatory markers by AVEE (e.g., NO, PGE2, TNF-*α*, and IL-1*β*) were inhibited by SnPP. These observations suggest that AVEE reinforces the Nrf2/HO-1 pathway and promotes antioxidant status in macrophages challenged by LPS. Furthermore, the above-mentioned results were wholly supported by the results obtained in our *ex vivo* study conducted using macrophages harvested from mice peritoneal cavity.

In a pioneering study, sulforaphane was found to suppress LPS-induced inflammation via the Nrf2 pathway in mouse peritoneal macrophages [47], and a large number of natural compounds have been reported to activate Nrf2 [48]. Though we elucidated the effects of AVEE on the Nrf2/HO-1 pathway, we did not identify the compound responsible in AVEE. Nevertheless, HPLC showed AVEE contained quercetin, which has been suggested to be an activator of the Nrf2/HO-1 pathway [49]. However, *A. villosum* has also been reported to contain several potential Nrf2 activators (e.g., bornyl acetate, camphor, borneol, *β*-sitosterol, and vanillic acid) [31]. Thus, we suggest further study be undertaken to identify the compound primarily responsible for the effects of AVEE observed in the present study.

## 5. Conclusion

Our findings show AVEE suppresses the NF-*κ*B signaling pathway and subsequent inflammatory responses by activating the Nrf2/HO-1 pathway and, thus, reduces oxidative stress in murine macrophages. We tentatively suggest that quercetin in AVEE is responsible for these beneficial effects. Further study is required to assess effects of AVEE on ROS-related chronic inflammatory diseases *in vivo*.

## Data Availability

All data used to support the findings of this study are included within the article.
